# First Complete Genome Sequence of *Felis catus* Gammaherpesvirus 1

**DOI:** 10.1128/genomeA.01192-15

**Published:** 2015-11-05

**Authors:** Ryan M. Troyer, Justin S. Lee, Momchilo Vuyisich, Patrick Chain, Chien-Chi Lo, Brent Kronmiller, Shay Bracha, Anne C. Avery, Sue VandeWoude

**Affiliations:** a Department of Biomedical Sciences, Oregon State University, Corvallis, Oregon, USA; b Department of Microbiology, Immunology and Pathology, Colorado State University, Fort Collins, Colorado, USA; c Los Alamos National Laboratory, Los Alamos, New Mexico, USA; d Center for Genome Research and Biocomputing, Oregon State University, Corvallis, Oregon, USA; e Department of Clinical Sciences, Oregon State University, Corvallis, Oregon, USA

## Abstract

We sequenced the complete genome of *Felis catus* gammaherpesvirus 1 (FcaGHV1) from lymph node DNA of an infected cat. The genome includes a 121,556-nucleotide unique region with 87 predicted open reading frames (61 gammaherpesvirus conserved and 26 unique) flanked by multiple copies of a 966-nucleotide terminal repeat.

## GENOME ANNOUNCEMENT

*Felis catus* gammaherpesvirus 1 (FcaGHV1) is a newly identified virus which clusters phylogenetically with members of genus *Percavirus*, subfamily *Gammaherpesvirinae*, family *Herpesviridae* (1). FcaGHV1 DNA has been detected in the blood of domestic cats in the United States, Australia, Singapore, and Central Europe (1–3). Infection is most common in adult males, cats coinfected with pathogenic retroviruses, and cats with poor health condition (2).

Using an FcaGHV1-specific real-time quantitative PCR (qPCR) (1), we detected a high level of viral DNA (48 FcaGHV1 genomes per cell) in archived mesenteric lymph node DNA from a 9-year-old male cat with intestinal T cell lymphoma from Florida, USA (sample 31286). To sequence FcaGHV1 directly from the lymph node DNA, we generated paired-end 150-nucleotide reads using an Illumina MiSeq. Sequences were assembled *de novo* using MIRA (4) as well as SPAdes 3.0 (5). Contigs were extended using MeGAMerge (6) and PRICE (7) to produce a 111-kb contig corresponding to the left end of the genome and a 6-kb contig corresponding to the far right end of the genome. PCR amplification and Sanger sequencing were used to connect the contigs and confirm regions containing repeats. Genome ends and terminal repeats were determined by genome walking using an APAgene GOLD genome walking kit (Bio S&T) and PCR amplification with a Roche GC-RICH PCR system. We identified multiple copies of a 966-nucleotide terminal repeat sequence on each end of the genome, but the exact number of repeats was not determined. The published sequence includes one copy of this terminal repeat sequence. We verified the final sequence by reassembly of the MiSeq reads to the consensus genome using Bowtie2 (8).

We defined open reading frames (ORFs) by prediction with GeneMarkS (9) and FGENESV (SoftBerry) and comparison to herpesvirus and cellular genes using NCBI BLAST, considering only ORFs encoding greater than 55 amino acids. In total we predicted 87 ORFs. Of these, 61 had homology to GHV conserved genes and were named based on the common herpesvirus saimiri ORF numbering system. The remaining 26 unique FcaGHV1 ORFs were named beginning with the letter “F” and numbered sequentially across the genome (F1—F26). These ORFs have little homology to the unique ORFs encoded by other fully sequenced percaviruses, equine herpesviruses (EHV) 2 and 5 (10, 11). An exception is F12, which has homology to EHV2 ORF E4. Like other gammaherpesviruses (GHVs), FcaGHV1 encodes viral homologs of cellular genes, including apoptosis regulators vFLIP (F7) and vBcl-2 (F9); the chemokine vCCL20 (F15); a homolog of Kaposi’s sarcoma-associated herpesvirus ORF K3 (F10), which downregulates MHC-I (12); a homolog of equilibrative nucleoside transporter 1 (F22); and vFGAM synthase (F18), which is commonly encoded by herpesviruses. The remaining 19 ORFs have no clear homology to known genes. A common feature of unique ORFs at the left end of the genome (F1, F2, F3, and F5) is the presence of relatively large repetitive sequences (266 to 1,706 nucleotides) within each ORF.

### Nucleotide sequence accession number.

The FcaGHV1 genome sequence was deposited in GenBank under accession no. KT595939.
